# Individualized antisense oligonucleotides for *SCN2A-*related developmental epileptic encephalopathy

**DOI:** 10.1038/s41591-026-04527-y

**Published:** 2026-07-21

**Authors:** Olivia Kim-McManus, Laurence Mignon, Julie Douville, He Pu, Catherine Parisien, Sarah Glass, C. Frank Bennett, Janelle Celso, Kendall Robbins, Hoameng Ung, Heather Olson, Stephen F. Kingsmore, Steven Petrou, Stanley T. Crooke, Joseph G. Gleeson, Elizabeth Berry-Kravis

**Affiliations:** 1 Department of Neurosciences, University of California, San Diego, La Jolla, CA USA; 2 Rady Children’s Institute for Genomic Medicine, San Diego, CA USA; 3 n-Lorem Foundation, Carlsbad, CA USA; 4 Ionis Pharmaceuticals, Carlsbad, CA USA; 5 Rush University Medical Center, Chicago, IL USA; 6 Boston Children’s Hospital, Harvard University, Boston, MA USA; 7 The Florey Institute of Neuroscience and Mental Health, University of Melbourne, Parkville, Victoria Australia

**Keywords:** Epilepsy, Antisense oligonucleotide therapy, Gene expression, Stem-cell biotechnology, Autism spectrum disorders

## Abstract

*SCN2A* variants are among the most common genetic causes of developmental and epileptic encephalopathies (DEEs), which can present with uncontrolled seizures at birth and account for 1–2% of all epileptic encephalopathies. A substantial fraction of causal variants are gain-of-function or mixed-function variants associated with increased channel open probability or greater sodium current flux. Here two parallel *n* = 1 clinical studies were conducted in two patients (9-year-old and 14-year-old boys) with *SCN2A*-related DEE. Individualized allele-selective antisense oligonucleotides (ASOs) were designed to target heterozygous intronic single-nucleotide polymorphisms (SNPs) for decreased expression of mutant *SCN2A* transcript while preserving the wild-type copy. Primary endpoints included quantitative change from baseline in seizure frequency and neurodevelopment, including motor scores. Efficacy measures were also individualized to each patient’s phenotype, including refractory seizures, developmental delay, autism spectrum disorder, choreoathetosis and gastrointestinal dysfunction. Patients experienced a reduction in seizure frequency (26% and 90% in the two patients, respectively), decreased use of concomitant medications and improvement in neurodevelopmental skills. Both ASOs were well tolerated, with no ASO-related serious adverse events. Continued long-term follow-up of these preliminary positive safety and efficacy findings is needed to confirm the disease-modifying potential of these ASOs. Haplotype phasing in a separate cohort of infants with *SCN2A*-related disorder (*SCN2A*-RD), diagnosed by rapid whole-genome sequencing, identified 16% of patients with compatible SNPs. These data provide a pathway from *n* = 1 to *n* of more patients with *SCN2A*-RD and other monogenic disorders. ClinicalTrials.gov registration: NCT06314490.

## Abstract (web summary)

In two patients with SCN2A epileptic encephalopathy, treatment with personalized allele-selective antisense oligonucleotides led to a decrease in seizure frequency with a positive safety profile.

## Main

Epilepsy is defined as a disorder of the brain characterized by aberrant, excessive neuronal hypersynchrony and an enduring predisposition to epileptic seizures, as defined by the International League Against Epilepsy^1^. Thus, channelopathies, which mechanistically mediate neuronal networks, have emerged as a critical cause of developmental and epileptic encephalopathies (DEEs), with over 900 identified monogenic etiologies^2^. DEEs are also associated with a high burden of abnormal epileptiform abnormalities on electroencephalography (EEG), a sign of aberrant neuronal connectivity that results in significant neurodevelopmental delay, and are associated with increased incidence of sudden unexpected death in epilepsy^2–4^. DEEs represent one of the most severe chronic neurological disorders of childhood, requiring complex specialty medical care, frequent emergency room visits and hospitalizations, with consequential socioeconomic impact^2–4^.

DEE11 (MIM 613721) is a severe neurodevelopmental disorder caused by gain-of-function (GOF) and mixed-function variants in the *SCN2A* gene, encoding the neuronal sodium channel Na_V_1.2 α-subunit. Specific biophysical changes, including alterations in voltage sensing and ion flux, result from individual missense variants, depending on the location and amino acid substitution^2^. *SCN2A* GOF variants increase open probability and current flux, resulting in pathophysiologic electrophysiologic changes and neuronal hyperexcitability, with an increased predisposition to seizures early in life^4,5^. Heterologously expressed *SCN2A* variants such as p.Arg853Gln exhibit a mixed GOF and loss-of-function (LOF) phenotype due to multiple defects in time-dependent and voltage-dependent channel properties, including enhanced slow inactivation^6^. LOF variants in *SCN2A* are typically associated with autism spectrum disorder (ASD) and intellectual disability with or without epilepsy; thus, there is dose sensitivity to the balance of *SCN2A* channel function^5–7^. The phenotypic spectrum in *SCN2A*-related disorders (*SCN2A*-RDs) encompasses intractable epilepsy, profound developmental impairment, communication and behavioral issues, movement disorders, ASD and associated morbidity^5–7^. Current treatment options for DEE11 are symptomatic with sodium channel-blocking antiseizure medications (ASMs) that are often ineffective. Because *SCN2A* haploinsufficiency is one of the commonest genetic causes of autism^5–7^, allele-selective ASO design is an important consideration for genetic intervention in GOF and mixed GOF/LOF *SCN2A* variants.

Antisense oligonucleotides (ASOs) have emerged as effective disease-modifying therapies for several neurological disorders, such as spinal muscular atrophy and amyotrophic lateral sclerosis, and provide an opportunity to selectively correct the effects of pathogenic DNA variants. In an *SCN2A* GOF mouse epilepsy model, an *SCN2A* gapmer ASO downregulated mutant GOF transcripts, reduced seizures and extended lifespan^8^. A nonallele-selective ASO reduced seizures in a premature infant with early-onset *SCN2A*-RD^9^. While promising, these strategies nonselectively target both gene copies, which in some patients may have undesired consequences from the reduction of both reference and pathogenic transcripts. In contrast, allele-selective gapmer ASOs use specific single-nucleotide polymorphisms (SNPs) to distinguish mutant from reference haplotypes, selectively reducing the target RNA transcript with minimal effect on expression of the healthy copy^10^. Such ASOs are often developed for an individual patient, as the ASO design strategy is highly specific to the sequence of each haplotype. While individualized, allele-selective ASOs are relatively newly described as drugs^10,11^, diagnoses of toxic GOF and mixed-function single-gene variants in DEEs have allowed use of these drugs on larger scales than previously thought possible. These ASOs are attractive for conditions in which nonselective targeting could produce untoward risk, especially for essential genes like *SCN2A* that display dosage sensitivity.

## Results

### Clinical characteristics

Patient 1 was a 9-year-old male with a heterozygous *SCN2A* GOF pathogenic variant (c.5645G>A, p.Arg1882Gln) and a history of neonatal-onset seizures. He had severe intellectual disability and ASD. He was nonverbal, could communicate with an assistive communication board and had good gross motor skills with a normal gait. He had seizures medically refractory to >10 ASMs with ~30 seizures per month at prestudy baseline, requiring up to three rescue medications administered twice a week to stop seizures. He had recurrent status epilepticus requiring emergency department visits or hospitalizations one to two times per year. He was treated with cenobamate and phenytoin and required high phenytoin levels since infancy for sodium channel blockade, with recurrent episodes of status epilepticus at baseline, often when serum phenytoin levels fell below 20 mg dl^−1^.

Patient 2 was a 14-year-old male with a heterozygous pathogenic mixed GOF/LOF *SCN2A* variant (c.2558G>A, p.Arg853Gln)^12^ and a history of infantile spasms beginning at 8 months. He had a history of multiple seizure types, including myoclonic, focal and tonic seizures, which were medically refractory to more than ten different ASMs. He was referred to hospice at 2 years of age with near-daily seizures. He had severe global neurodevelopmental delay and was nonverbal, but had the ability to communicate using an assistive communication board. He was nonambulatory with prominent choreoathetosis and dyskinesias. He had debilitating chronic gastrointestinal issues, regularly requiring suppositories to initiate bowel movements.

A description of the individual genotypes and phenotypes of each patient is provided in Table 1.

**Table 1:** *SCN2A* genotype–phenotype heterogeneity of trial participants

Age, sex	Patient 1	Patient 2
	9-year-old male	14-year-old male
Mutation	Pathogenic, dominant GOF de novo variant in *SCN2A* (c.5645G>A, p.Arg1882Gln)	Pathogenic, mixed GOF/LOF de novo variant in *SCN2A* (c.2558G>A, p.Arg853Gln)
Age at seizure onset	Neonatal onset (at birth)	8 months of age (<1 year)
Seizure type	Focal, generalized tonic-clonic	Tonic, myoclonic, infantile spasms
Concomitant seizure medications	Phenytoin, cenobamate	Cannabidiol, clonazepam
Number of ASMs tried before the ASO trial	>10	>10
Communication	Nonverbal (can use AAC device)	Nonverbal (can use AAC device)
Behavioral	ASD, stereotyped and repetitive behaviors, irritability, abnormal sensory-seeking behaviors	ASD, irritability
Muscle tone	Normal	Axial hypotonia with appendicular hypertonia, GMFCS level V at baseline
Movement disorder, gait issues	History of dystonia; normal gait with intact running and jumping at baseline	Nonambulatory at baseline; significant choreoathetosis
Gastrotomy tube (Y/N)	N	Y
Scoliosis (Y/N)	N	N
Gastrointestinal issues (Y/N)	Y	Y (severe)

AAC, augmentative and alternative communication; GMFCS, Gross Motor Function Classification; Y, yes; N, no.

### Primary outcomes: seizures

**Fig. 1:**
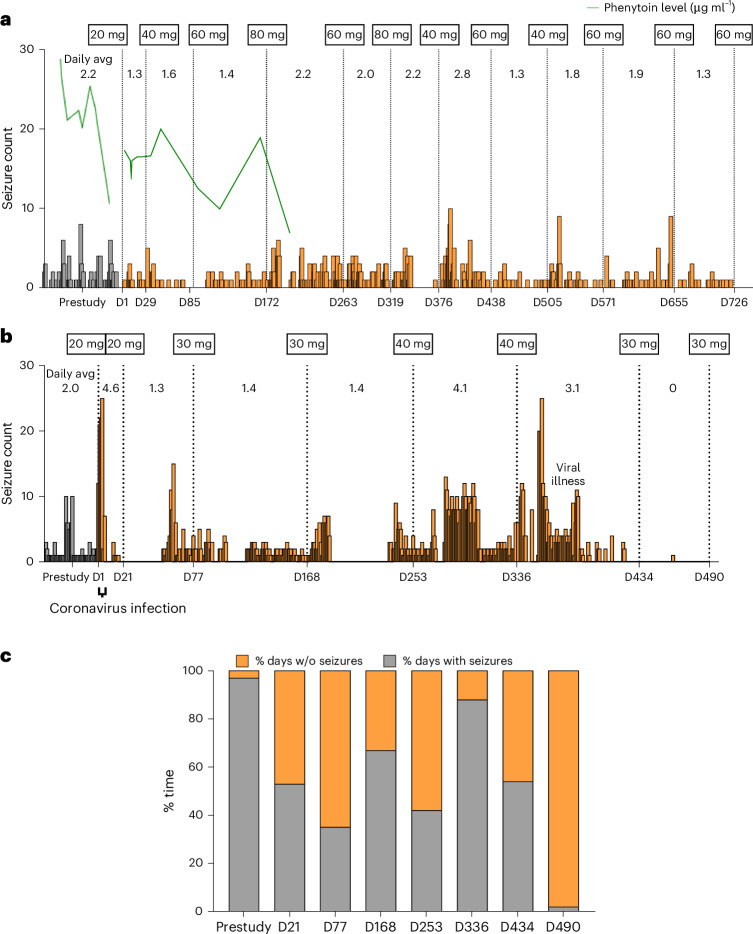
Analysis of changes in daily CMSF across time. **a**, Decrease in Patient 1 CMSF is observed since ASO initiation. To date, Patient 1 has received 12 doses of ASO over 24 months. His pre-ASO daily average seizure count (gray represents pre-ASO, orange represents post-ASO) was 2.2, which reduced to 1.3–1.6 in the first 6 months of treatment (D1–D172) and between 1.3–1.9 in the last 9 months (since D438). Of note, the patient previously required high doses of phenytoin since birth, which was weaned off ~day 310 (green line represents phenytoin levels, range 6.9–33.1 µg ml^−1^, with no further levels checked toward the end of the wean), suggesting ASO efficacy with reduced sodium channel-blocker requirement. **b**, Decrease in Patient 2 CMSF observed since treatment initiation. **c**, To date, Patient 2 has received eight doses over 16 months, with a 2.0 daily average seizures pretreatment to 0 daily average seizures after 14 months of treatment. CMSF, countable motor seizure frequency; w/o, without; D, day; avg, average.

Both patients showed improvements in seizure control, including prolonged periods of seizure freedom and reduction in concomitant medications (Fig. 1a,b).

Patient 1’s seizures were recorded consecutively for 90 days in the pre-ASO period and 744 days in the post-ASO period. He experienced improvement in seizure control with <80 mg doses and a dosing interval of 60–75 days. The dosing interval was shortened due to recurrent seizures toward the end of the 90-day dosing interval. On prespecified testing, the percentage of seizure-free days in the post-ASO period improved from 57.8% to 66.5% and corresponded to an estimated 26% reduction in seizure counts, but this did not meet statistical significance (*β* = −0.298, 95% confidence interval (CI) = −0.846 to 0.249, *P* = 0.286). It is noted, however, that he was able to be weaned off phenytoin around day 310, a sodium channel blocker with substantial side effects on which he had been dependent since birth for seizure control (Fig. 1a,b).

Patient 2’s seizures were recorded consecutively for 42 days in the pre-ASO period and 480 days in the post-ASO period. He experienced seizure-free days early in treatment (after 20 mg and 30 mg) but had bouts of seizures at 40 mg dosing. Due to waning efficacy seen 60 days post dosing, the dosing interval was decreased to 60–90 days, resulting in longer periods of seizure control without emergency seizure rescue medications for >2-month intervals (Fig. 1c). Seizures after initiation of ASO were significantly reduced by an estimated 90% in prespecified statistical analysis (*β* = −2.338, 95% CI = −3.021 to −1.655, *P* < 0.001). Similarly to Patient 1, the percentage of seizure-free days increased, from 0% in the pre-ASO period to 46% in the post-ASO period (Fig. 1c). For the prespecified statistical analysis of seizures, generalized linear models were optimized separately for each patient to evaluate daily seizure counts as a function of time, treatment status and ASO dose. A 1-day lag term was included for seizure counts to account for the time correlation of seizures. A negative binomial generalized linear model with a log-link function was used to account for overdispersion. All analyses were conducted separately for each patient.

For Patient 1, the model explained a modest proportion of variance (pseudo-*R*^2 ^= 0.176, dispersion statistic = 1.47). The treatment effect was not statistically significant (*β* = −0.298, 95% CI = −0.846 to 0.249, *P* = 0.286). The lagged seizure term was a strong predictor (*β* = 0.3171, 95% CI = 0.261 to 0.373, *P* < 0.001), indicating short-term autocorrelation, an expected finding due to repeated measures over time. Neither the time trend (*β* = −0.0004, 95% CI = −0.0010 to 0.000086, *P* = 0.122) nor ASO dose (*β* = 0.0042, 95% CI = –0.003 to 0.012, *P* = 0.272) was significant.

For Patient 2, the model explained a greater proportion of variance (pseudo-*R*^2 ^= 0.616, dispersion statistic = 1.49). The treatment effect was highly significant (*β* = −2.338, 95% CI = −3.021 to −1.655, *P* < 0.001), corresponding to an estimated 90% reduction in expected daily seizures in the post-ASO period. A significant time trend was observed (*β* = −0.0053, 95% CI = −0.007 to −0.004, *P* < 0.001), and the lag term again indicated strong autocorrelation (*β* = 0.199, 95% CI = 0.178 to 0.219, *P* < 0.001). Interestingly, while ASO administration corresponded to seizure reduction, higher ASO dosing was associated with slightly increased seizures compared to lower ASO doses for this patient (*β* = 0.105, 95% CI = 0.078 to 0.131, *P* < 0.001). This suggests the need for continued long-term follow-up.

Both patients had sustained seizure-free periods of consecutive weeks to months, and neither had seizure-related emergency room visits or hospital admissions after ASO initiation.

### Secondary outcomes: neurodevelopmental

**Fig. 2:**
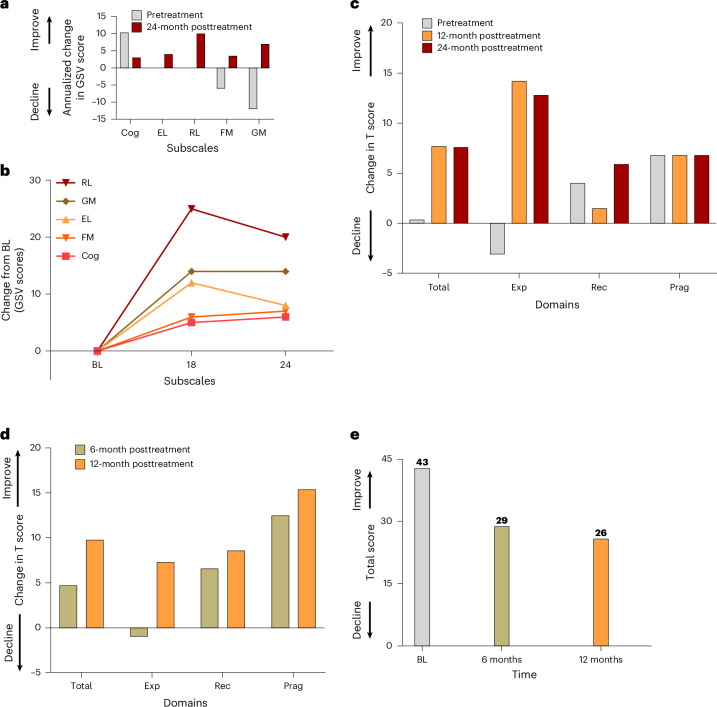
Analysis of changes in neurodevelopmental and communication measures across time. Improvements in neurodevelopmental skills were seen across multiple outcome domains in both patients. **a**, Annualized rate of change in different BSID-4 domains pretreatment and posttreatment, showing improvement in rate in all domains except cognitive. Fourteen months of pretreatment data were used for cognitive and expressive language subscales; four months of pretreatment data were used for fine and gross motor subscales. No pretreatment data were available for the receptive language subscale. **b**, Actual GSV change from baseline over time. For Patient 1, improvements in GSVs were observed across all subscales, with an improved annualized growth rate in some domains on the BSID-4. **c**, ORCA change from baseline pretreatment and posttreatment in Patient 1. Patient 1 also showed clinically meaningful improvement on ORCA. **d**, ORCA change from baseline in Patient 2. Patient 2 also had clinically meaningful changes in ORCA. This suggests an increased rate of development in language and motor skills, where slowing of the cognitive growth rate with age and development would typically be expected. Aberrant, stereotyped and abnormal sensory behaviors have also significantly improved. **e**, D-FIS at baseline and over time. Patient 2 had a reduction in ataxia and improvement in motor ability, including the developmental milestone of independent gait at 15 years of age. Cog, cognitive; EL, expressive language; RL, receptive language; FM, fine motor; GM, gross motor; Exp, expressive; Rec, receptive; Prag, pragmatic; BL, baseline; D-FIS, Dyskinetic Cerebral Palsy Functional Impact Scale.

**Fig. 3:**
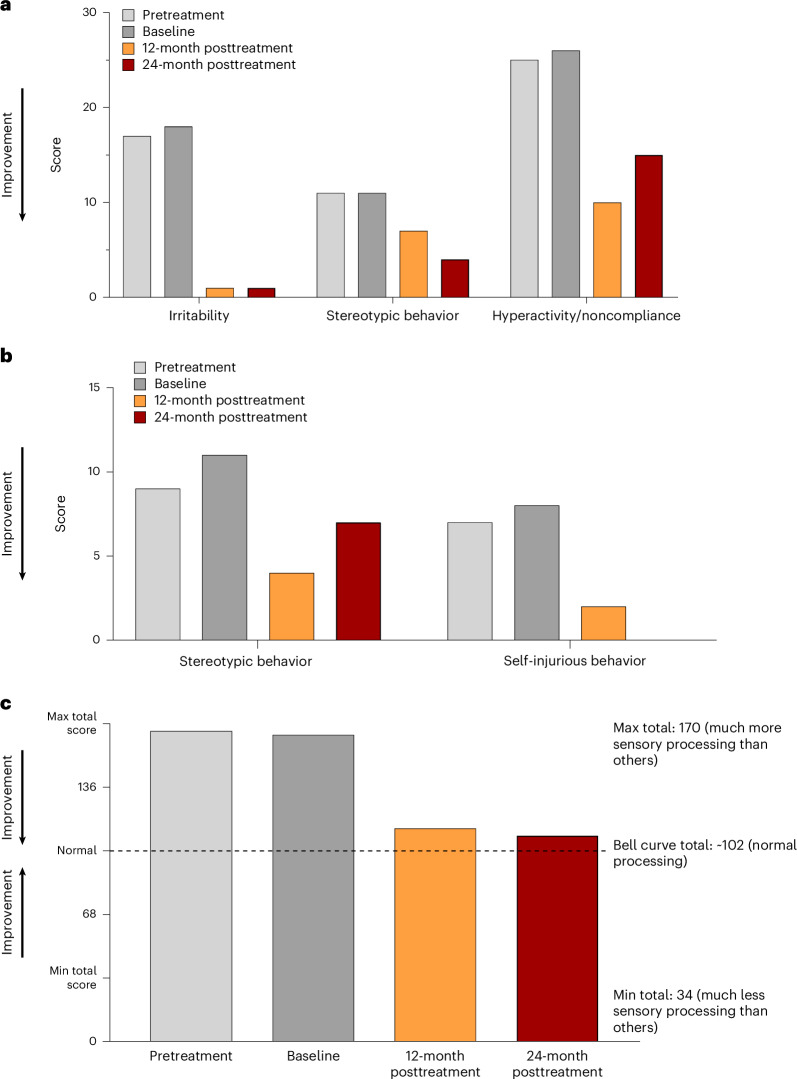
Additional endpoints in Patient 1. **a**,**b**, The RBS-R includes six domains—ritualistic behavior, sameness behavior, stereotypic behavior, self-injurious behavior, compulsive behavior and restricted behavior. Patient 1 had improvements in irritability and hyperactivity (**a**), stereotyped and self-injurious behavior (**b**). **c**, The SSP-2 is a standardized questionnaire designed to help evaluate a child’s sensory processing patterns, including overarousal and hypersensitive behaviors in home, school and community settings. Patient 1 had improvement in sensory processing. RBS-R, Repetitive Behavior Scale-Revised; SSP-2, Short Sensory Profile-2; max, maximum; min, minimum.

Changes in neurodevelopmental skills were seen across multiple outcome domains in both patients. For Patient 1, there were improvements in Growth Scale Values (GSVs) across all subscales, with an improved annualized rate of growth in some domains on the Bayley Scales of Infant and Toddler Development, Fourth Edition (BSID-4; Fig. 2a,b). Patient 1 also showed clinically meaningful improvement on Observer-Reported Communication Ability (ORCA; Fig. 2c), suggesting an increased rate of development in language and motor skills, where slowing of cognitive growth rate with age and development would typically be expected. Aberrant, stereotyped and abnormal sensory behavior also improved (Fig. 3a–c).

**Fig. 4:**
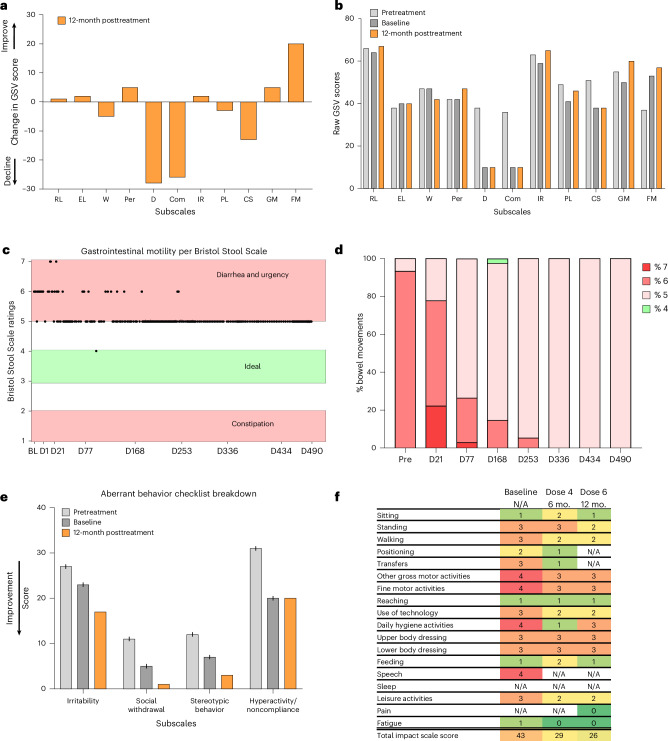
Additional endpoints in Patient 2. **a**,**b**, Vineland-3 Adaptive Behavior Scales. The Vineland-3 is a valid and reliable measure of adaptive functioning from birth to adulthood. In addition to standard scores and age equivalents, GSVs are available for all subdomain scores, allowing reliable assessment of change even in those who floor the measure or appear to make no progress when using standard scores due to a functional level far below chronological age. **a**, Change from baseline in GSV scores 1 year posttreatment. **b**, GSV scores across three time points (pre-treatment, baseline and 1 year posttreatment). **c**,**d**, Bristol Stool Form Scale. The Bristol Stool Form Scale is a diagnostic medical tool designed to classify the form of human feces into seven categories. It has been used to track and diagnose irritable bowel syndrome and is sensitive to changes in intestinal transit time following several medications. **c**, As shown post-ASO, Patient 2 showed improvement in bowel movements, specifically with respect to irritable bowel and intestinal transit. A score of 3–4 on this scale represents normal bowel movements, while 7 is indicative of diarrhea, and 1 is indicative of constipation. **d**, Patient 2 had more normalized bowel movements over time (sustained score of 5) and required minimal suppository use. **e**, ABC-C. The ABC-C is a 58-item parent/caregiver rating scale used to assess the severity of a range of problem behaviors commonly observed in individuals with intellectual disability across five dimensions or subscales—irritability, hyperactivity, lethargy/withdrawal, stereotypy and inappropriate speech. At 12 months posttreatment, Patient 2 exhibited improvements in irritability, social withdrawal and stereotyped behavior. **f**, D-FIS. The D-FIS is a parent-reported measure used to assess the impact of abnormal muscle tone, such as spasticity (increased tone or stiffness), floppiness (decreased tone) and dyskinesia (fluctuating tone) on the patient’s ability to perform daily activities. Over time, Patient 2 showed improvement in different domains of the D-FIS scale. W, written; Per, personal; D, Domestic; Com, community; IR, interpersonal relationships; PL, play and leisure; CS, coping skills; Pre, pretreatment/baseline period; N/A, not applicable; mo., months; ABC-C, Aberrant Behavior Checklist-Community Edition.

Patient 2 demonstrated clinically meaningful changes in communication (Fig. 4a,b) and sustained trends toward reduction of maladaptive behaviors and irritability (Fig. 4e). Patient 2 also had a remarkable reduction in ataxia and improvement in motor ability, including the notable developmental motor milestone of independent gait at 15 years of age (Figs. 2e and 4f).

### Other domains

**Extended Data Fig. 1:**
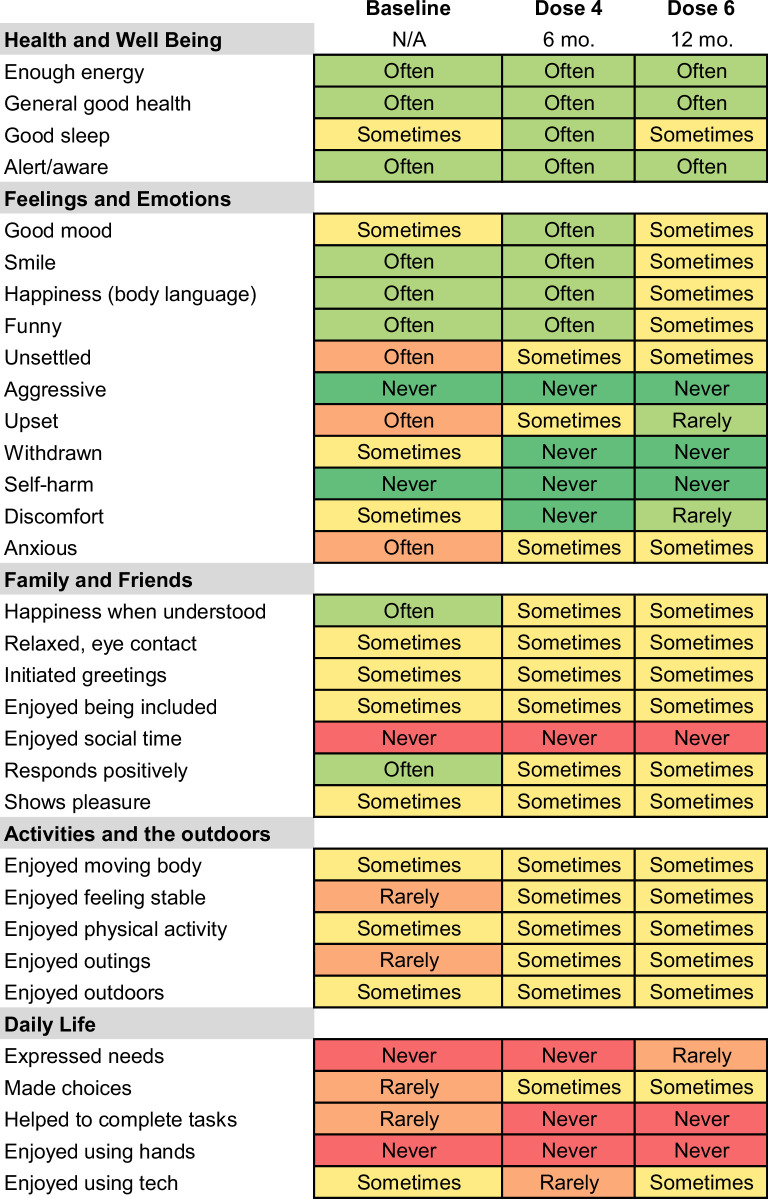
Quality of Life-Disability (QOL-D). The Quality-of-Life Inventory–Disability (QOL-D) is a 32-item parent/caregiver rating scale to assess the child’s quality of life over the previous month based on the following domains: health and well-being, feelings and emotions, family and friends, activities and the outdoors and daily life. Patient 2 showed a trend towards improved quality of life post ASO in multiple scale domains.

Improvement was observed across other domains. Patient 2 had required frequent suppositories for severe gastrointestinal symptoms, which were tracked with the Bristol Stool Form Scale (Fig. 4c,d). Post-ASO, Patient 2 required minimal suppository use, with bowel movements normalizing, suggesting that ASO treatment may have positively modulated autonomic dysfunction. Patient 2’s quality-of-life and disability scores showed sustained improvements across multiple domains over time (Extended Data Fig. 1).

### Safety

**Extended Data Fig. 2:**
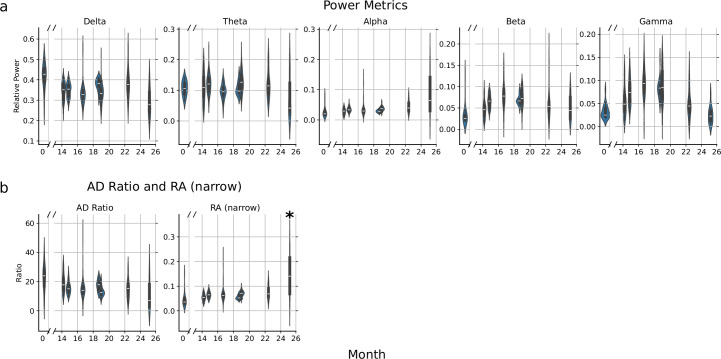
Electroencephalography (EEG) acquisition and analysis. Additional EEG spectral analysis was conducted to evaluate for baseline changes in frequencies in Patient 2. EEG data were collected using Natus and Nihon Kohden systems, with session 0 as a baseline and after ASO administration. Recordings used a standard international 10–20 montage at 256 Hz (Natus) or 200 Hz (Nihon Kohden) sampling rate. The resulting signals were bandpass filtered (1–70 Hz, FIR) and notch filtered at 60 Hz to attenuate line noise. Each EEG was manually reviewed by Board-certified epileptologists and significant artifacts as well as sleep, were annotated and removed from analysis. Spectral features were computed from non-overlapping 5-minute epochs using Welch’s method. EEG spectral power was calculated across 5-minute epochs for each session. Metrics included relative power in canonical frequency bands: delta (1–4 Hz), theta (4–8 Hz), alpha (8–13 Hz), beta (13–30 Hz), and gamma (30–50 Hz) (**a**) as well as alpha/delta (AD) ratio and alpha power relative to a narrow band (RA (narrow), alpha power/(1–20 hz) (**b**)). Violin plots, which display the median, interquartile range, and 1.5× interquartile range along with a kernel density estimation, are shown. A mixed effects linear model was fit with each metric as the dependent variable and each 5 minute epoch nested within each session, with each session modeled as a random effect. The relative power as well as ADR and RA (narrow) values are shown with violin plots, which display the median, interquartile range, and 1.5× interquartile range along with a kernel density estimation. No significant difference was seen between the relative power in all frequency bands (**a**), nor is there a significant difference between the ADR (**b**). There is, however, a significant increase in RA (narrow) for the EEG collected after the last session* (β = 0.087, SE = 0.040, z = 2.20, p = 0.028, 95% CI (0.009, 0.165)) (**b**). The EEG frequency analysis suggests an increase in relative alpha at the last session, which may suggest an improvement in cortical function.

No ASO-related adverse events or serious adverse events were reported in either patient (Table 2). Both ASOs were well tolerated, with no abnormal biochemical or hematological laboratory results and no significant changes in electrocardiography (ECG) or EEG findings (Extended Data Fig. 2) compared with baseline throughout the study.

**Table 2:** All adverse events reported for both patients throughout study duration

MedDRA body system	Patient	Adverse event grade	Serious?	Related to study drug administration	Related to study drug
Endocrine disorders					
Growth hormone abnormal	1	Moderate	No	No	No
Gastrointestinal disorders					
Vomiting	1	Mild	No	No	No
Infections and infestations					
Bacterial infection	1	Mild	No	No	No
Viral infection	1	Mild	No	No	No
COVID-19 respiratory infection	1	Mild	No	No	No
Coronavirus	2	Mild	No	No	No
Common cold	2	Mild	No	No	No
Viral infection	2	Moderate	No	No	No
Injury, poisoning and procedural complications					
Vomiting postoperative	1	Mild	No	Definite	No
Wrist fracture	1	Moderate	No	No	No
Fall	2	Moderate	No	No	No
Fall	3	Moderate	No	No	No
Nervous system disorder					
Sleep restless	1	Mild	No	No	No
Feeling sad	1	Mild	No	No	No
Somnolence	1	Mild	No	No	No
Tic	1	Mild	No	No	Possible
Respiratory, thoracic and mediastinal disorder					
Epistaxis	1	Mild	No	No	No

### Additional potential patients

The allele-selective ASO developed specifically for both patients has the potential to be used in patients with other causal GOF or mixed *SCN2A* variants if they are heterozygous for the same SNP on the reference haplotype, as this could confer the needed selectivity to downregulate expression from the pathogenic haplotype.

We analyzed a cohort of 19 *SCN2A*-RD probands identified through rapid whole-genome sequencing (WGS) at the Rady Children’s Institute for Genomic Medicine between 2018 and 2024, diagnosed at ages ranging from 2 weeks to 12 years^13^. Sixteen percent (that is, three patients) in our cohort were identified as having the correct SNP configuration amenable to treatment with the same ASO initially designed for Patient 2, supporting the potential to enable use of reference sequence-targeting ASOs to additional patients. Patients with *SCN2A* variants of uncertain significance could be eligible for ASO therapy with functional characterization of missense and small in-frame insertion or deletion variants to establish GOF or mixed-function effects amenable to the ASO approach.

## Discussion

Individualized allele-selective ASOs for *SCN2A-*DEE11 variants with differing genotypes and phenotypes associated with significant morbidity reveal positive safety and efficacy signals with clinically meaningful improvements in seizures and across multiple neurodevelopmental domains. Improvements in seizure control can be achieved with traditional ASMs; however, our targeted approach with ASOs addresses the underlying root causes of the disease mechanism, with multiple efficacy points beyond seizures. Improvements were observed across developmental and motor skills and other domains beyond traditional ASMs. These results support that neurodevelopmental phenotypes may remain at least partially treatable by targeted genetic therapies beyond early development, as seen in ASO trials for Angelman syndrome^14^. Of note, the ASOs were effective for both GOF and mixed-function pathogenic variants and across distinct *SCN2A*-associated phenotypes, including DEE11 and ASD.

Dose optimization posed specific complexities due to concurrent use of sodium channel-blocking ASMs, the risk of seizure exacerbation from excessive sodium channel reduction, potential circuit-level effects and long-term neural adaptation to underlying pathophysiology.

These *n* = 1 trials required predefined individualized treatment goals to assess meaningful change based on the concept of the minimal clinically important difference (MCID), the smallest response considered clinically impactful^15^. MCID is difficult to define in rare populations, especially in *n* = 1 (refs. ^14,15^); however, improvements in these patients exceeded MCID cutoffs on the ORCA for other conditions and as well as thresholds used to define improvement in Angelman syndrome ASO trials on the Bayley-4 (refs. ^14,15^). Patients met prespecified outcomes by exceeding baseline annual rates of gain after treatment with ASOs. Outcome assessments were predefined for phenotype to assess change pre-ASO and post-ASO, including GSV scores, which can demonstrate meaningful clinical change in developmentally delayed individuals who may not be assessed on Bayley-4 against standard neurotypical scores. Patients demonstrated gains beyond typical developmental windows, providing substantial insights regarding evolving and effective trial design and implementation of outcome measures.

There may be limitations to individual ASO-related benefits for interventions in diseases where there is irreversible or limited potential to change the metabolome, proteome or neuronal networks unless implemented very early in life. In refractory early-onset epilepsy due to GOF variants, there may be a limited reduction in seizures due to the underlying pathophysiology. Early and accurate genetic diagnosis will enable optimization of the design and delivery of potential therapeutic neurointervention for maximal benefit to patients. Long-term follow-up will further inform the durability of safety and efficacy findings. Despite these limitations, research studies such as ours establish feasibility and tolerability, validate therapeutic platforms, and enable further understanding of which diseases are modifiable over very long-term follow-up and those which are not. In combination with rapid WGS, which allows genetic diagnosis of DEE11 within days of first seizure onset, targeted neurointerventional disease-modifying therapies have the potential not only to decrease seizures but also to impact neurodevelopment. Thus, our study demonstrates an important paradigm shift—that there may be complementary approaches even within a single monogenic condition (that is, *SCN2A*) with significant genotype–phenotype heterogeneity, providing therapeutic pathways for more patients with plausible mechanisms beyond GOF.

Open-label, first-in-human, investigational individualized allele-selective ASO therapy demonstrates positive safety and efficacy in two patients with different causal GOF and mixed GOF/LOF *SCN2A* variants and phenotypes, characterized by reduction in seizures and concomitant medications, increased periods of seizure freedom, decrease in autistic behaviors and improvement in neurodevelopmental outcomes with disease-modifying effect, offering an alternative treatment paradigm and enabling a pivotal pathway from ‘*n* = 1’ to ‘*n*-of-many’ more patients with *SCN2A*-RD and other rare monogenic disorders.

## Methods

### ASO discovery and design

Individualized ASOs were designed for two patients with intractable epilepsy with GOF and mixed GOF/LOF causal variants associated with DEE11. WGS identified SNPs on the reference haplotype, allowing the design of ASOs targeting only the pathogenic haplotype. A common benign SNP on the wild-type allele in each patient was used to develop a selective ASO for the mutant allele. These ASOs were designed to promote selective degradation of the mutant *SCN2A* transcript through recruitment of RNase H1 to the RNA–ASO heteroduplex^16,17^. Over 500 2’-methoxyethyl gapmers with mixed backbone (phosphorothioate/phosphodiester) were designed to single-nucleotide differences between the two alleles in the patients’ *SCN2A* gene. ASOs targeted either the SNP or the consensus sequence phased to the pathogenic allele as previously described^16^.

We mapped informative heterozygous SNPs using long-read sequencing in both Patient 1 and Patient 2. We were unable to identify a single ASO that could be used to treat both patients, so instead we identified individual ASOs for each patient. Multiple potent, allele-selective ASOs identified in a single-dose in vitro assay in each induced pluripotent stem cell (iPSC)-derived neuronal line were tested via dose–response assays. Excitatory neurons were generated from iPSCs using the Quick-Neuron Excitatory Sendai Virus (SeV) system (Elixirgen Scientific) following the manufacturer’s protocol.

**Extended Data Fig. 3:**
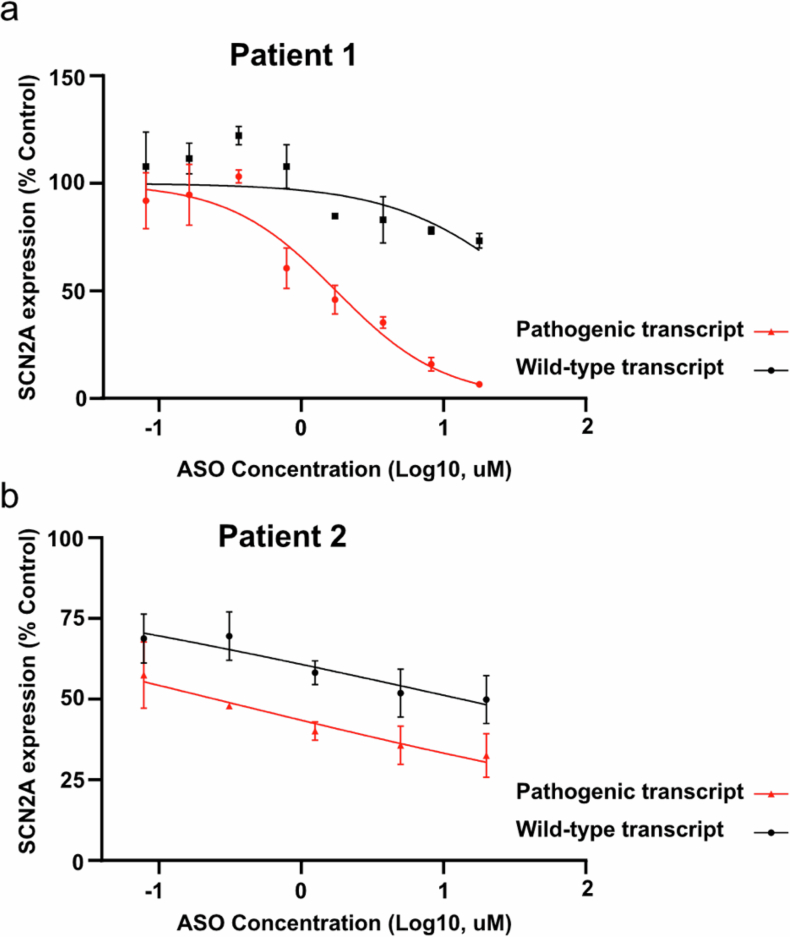
Dose-response curve of ASO for Patient 1 and Patient 2. In vitro potency and allele-selectivity determined by allele-selective ASO dose response in patient iPSC-derived neurons by free uptake at concentrations of 0.065 µM, 0.16 µM, 0.4 µM, 1.024 µM, 2.56 µM, 6.4 µM, 16 µM, and 40 µM (Patient 1; **a**) or 0.078, 0.3125, 1.25, 5, and 20 µM (Patient 2; **b**) for 5–7 days. Data are presented as mean values ± s.d. Cells were collected and RNA isolated using RNAeasy 96-well kit (Qiagen) according to manufacturer’s instructions. Expression of each allele was then measured using tagged probes (‘Wild-type transcript’: black; ‘Pathogenic transcript’: red). SCN2A mRNA was assessed using quantitative RT-PCR (TaqMan) assay on an Applied Biosystems 7900H Fast or QuantStudio 7 Real-Time PCR system. Allele selectivity was seen in both individualized ASOs; ASO demonstrated 42-fold selectivity for Patient 1 and 53-fold selectivity for Patient 2, based on IC50 value for mutant versus wildtype transcripts. The IC50 for Patient 1’s ASO was calculated to be 2.61 µM for the pathogenic transcript and greater than 40 µM for the wild-type transcript. The IC50 for Patient 2’s ASO was calculated to be 0.248 µM for the pathogenic transcript and 13.37 µM for the wild-type transcript.

**Extended Data Fig. 4:**
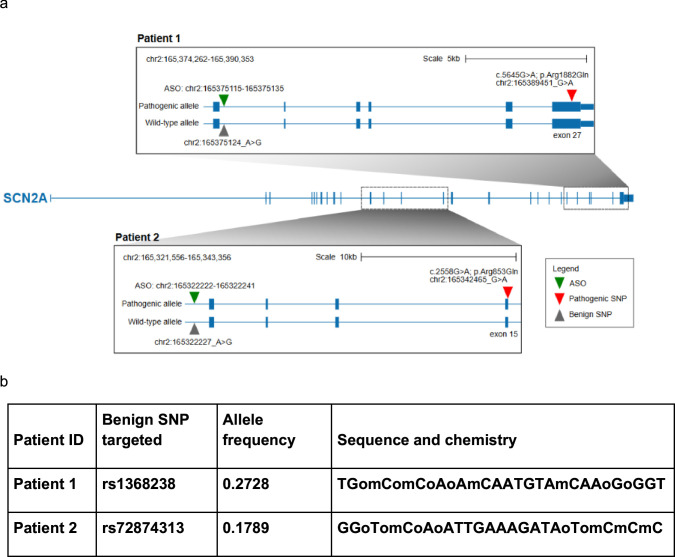
ASO design and ASO sequence and chemistry. **a**, Graphic representation of where the ASO binds relative to the pathogenic mutations, utilizing a benign SNP identified on the wild-type allele. **b**, Specific sequence and chemistry of the 2 allele-selective ASOs designed for Patient 1 and Patient 2. Allele frequency in the global population from 1000 Genomes as per https://www.ncbi.nlm.nih.gov/snp/rs1368238 and https://www.ncbi.nlm.nih.gov/snp/rs72874313/. Key for ASO chemical modifications: black, unmodified deoxyribose; blue, 2 methoxyethyl (MOE); unmarked backbone linkages, phosphorothioate (PS); linkages marked with o, normal phosphodiester (PO). mC, 5-methylcytosine.

A distinct lead ASO was identified for each patient and assessed for allele selectivity in patient iPSCs (Extended Data Fig. 3). Allele selectivity was observed for both individualized ASOs; the ASO demonstrated 42-fold selectivity in patient 1 and 53-fold selectivity in patient 2, based on IC_50_ values for mutant versus wild-type transcripts. The SNPs used for allele selectivity in Patients 1 and 2 had population allele frequencies of 0.27 and 0.18 (that is, 27% and 18% in most sampled populations), respectively, suggesting other patients could potentially be treated with these same ASOs in the future (Extended Data Fig. 4). The respective ASOs were assessed for potency and selectivity using in vitro assays in patient-derived iPSCs for inflammation and off-target vulnerability.

ASOs meeting minimal criteria for potency and selectivity were assessed for their potential to trigger innate immunity by measuring CCL22 levels in BJAB cells treated with high ASO concentrations (up to 8 µM) as described in ref. ^18^, followed by in vitro confirmation of allele specificity. In silico identification of human primary transcripts with partial complementarity to lead ASOs (1–2 mismatches or 3 mismatches with ≥17 consecutive matches) was assessed. Transcripts lacking expression in GTEx were excluded, and the remaining candidates were tested in a cell-based assay across several ASO concentrations. Of these, two genes (*ANKS1B* and *TXK*) showed dose-dependent reduction following treatment with nL-SCN2-001, while one (*CFAP47*) could not be evaluated due to a lack of expression in available cell lines. Thus, gene-specific assessment indicated a low off-target liability. *TXK* had no predicted LOF concern (gnomAD observed/expected (O/E) = 0.53, pLI = 0), showed no phenotype in knockout mice, suggesting reduction was tolerated. *ANKS1B* showed LOF constraint (O/E = 0.1, pLI = 1.0), but the knockout mouse was phenotypically normal. *CFAP47* was associated with infertility in male mice, but this risk was not considered relevant for intrathecal (IT) dosing. Overall, the combined in silico, in vitro and genetic evidence supported a low off-target safety risk for both ASOs.

ASOs were then assessed for in vivo tolerability in two non-Good Laboratory Practice studies (an 8-week single intracerebroventricular dose study in mice and an 8-week single IT dose study in rats)^19,20^. These steps led to the identification of an optimal ASO for each patient. The lead ASOs were then evaluated in two distinct 13-week Good Laboratory Practice repeat-dose toxicology studies in which 10 rats of each sex were given 0, 0.3 or 1 mg per dose by IT administration on Days 1, 29, 57 and 85, with necropsy performed on Day 92.

In both studies, the ASOs were considered to be well tolerated, with transient postdose clinical and neurobehavioral observations of abnormal gait, incoordination, limited use and/or decreased grip strength, low carriage, reduced arousal, decreased alertness, reduced rearing events, decreased body temperature, decreased body tone, impaired tail-pinch response/tactile reflex and/or decreased activity noted at 1 mg per dose, which recovered within 24 h. Microscopic findings were observed at ≥0.3 mg per dose and included vacuolated macrophages and mononuclear or mixed-cell infiltration in the spinal cord, injection site, meninges, nerve roots, brain, liver, dorsal root ganglion and/or kidney, as expected. In addition, basophilic granules and/or tubular degeneration/regeneration were noted in the kidney, and minimal-to-mild nerve fiber degeneration was observed in the spinal cord, nerve roots and/or injection site, as expected.

All findings were considered nonadverse and were similar to those observed in commercial ASOs, and the no-observed-adverse-effect level was established at 1 mg per dose for both ASOs.

### Patients and study design

Research investigational new drug applications were authorized by the FDA for investigator-initiated, open-label, single-center, single-patient (*n* = 1) clinical studies of distinct pathogenic *SCN2A* variants with predefined safety and efficacy measures tailored to individual phenotype.

The research studies were approved by experimental treatment ethics committees and institutional review boards (IRBs) of the respective academic institutions. Written informed consent or assent, including reporting of indirect identifiers, was obtained from study participants’ legally authorized representatives before study initiation according to CARE guidelines and in compliance with the Declaration of Helsinki principles. Rush University IRB approval was received under compassionate use in May 2023, and consent and enrollment occurred in June 2023 (Rush ORA 2305010), with 24 months of follow-up until the data cutoff in June 2025. UCSD Rady IRB approval was received in November 2023, and consent and enrollment occurred in February 2024 (UCSD NCT06314490), with 16 months of follow-up at the data review in June 2025.

The allele-selective ASOs were delivered intrathecally by lumbar injection in these two first-in-human clinical trials. Each trial was customized to the patient-specific *SCN2A*-RD phenotype, with predefined outcome measures assessing seizures, behavior, communication, gastrointestinal issues, dyskinesias and motor skills.

Inclusion criteria for both patients included confirmation of a causal *SCN2A* variant, informed consent or assent, stable dosing of concomitant medications, the ability to travel to the study site and adhere to study-related procedures and the ability to notify the research team of any adverse events. Additional inclusion criteria for Patient 1 included refractory epilepsy with at least four seizures per month on daily ASM.

Exclusion criteria for both patients included use of other investigational medications within five half-lives at study enrollment and contraindicated conditions to safe or effective IT lumbar puncture or related sedation beyond standard risk (for example, thrombocytopenia or other bleeding diathesis, space-occupying intracranial lesions, or infection of skin or subcutaneous tissues near the lumbar puncture site). Additional exclusion criteria included any comorbid condition that, in the opinion of the respective investigators, would prevent completion of study procedures or exacerbate the patient’s underlying condition (for example, seizures).

Following baseline assessments, ASOs were dose-escalated according to each individualized protocol schedule of activities. Each patient-specific protocol was amended in response to FDA feedback to optimize dosing based on emerging clinical data, with regulatory-driven refinement of endpoints and dosing strategy. For both patients, early amendments refined dose-escalation criteria for seizure-related outcomes and allowed flexible dosing intervals (60–90 days), with criteria for shortening intervals based on waning seizure control and rescue medication use. For Patient 2, additional early amendments expanded the primary endpoints beyond seizure frequency to include assessments of motor and gastrointestinal domains. Later amendments incorporated additional outcome measures due to patient improvement and developmental skills gained (including gait). Dosing interval flexibility (60–90 days) was authorized to incorporate multidomain clinical criteria (seizure, motor and gastrointestinal changes) in guiding dosing interval adjustments. All amendments were approved by the FDA and local IRBs before implementation. There were no protocol deviations in either clinical trial, which remain active at their respective academic institution.

### Primary endpoints

For both patients, primary endpoints were predefined as the change from baseline in countable motor seizure frequency at 12 and 24 months after ASO administration. Statistical analysis of seizure counts was conducted using a generalized linear model.

Additional primary endpoints for Patient 2 included change in neurodevelopmental scores from baseline at 12 and 24 months after initiation of ASO, including movement and motor domain scores on the Vineland Adaptive Behavior Scales–Version 3 (Vineland-3), BSID-4 with GSVs, and Dyskinetic Cerebral Palsy Functional Impact Scale (D-FIS). Outcome assessments were predefined for phenotype to assess change pre-ASO and post-ASO, including GSV scores, which can demonstrate meaningful clinical change in developmentally delayed individuals who may not be assessed on Bayley-4 against standard neurotypical scores.

The primary endpoint for Patient 2 also included the change in score from baseline at 12 and 24 months post-ASO on the Bristol Stool Form Scale as a surrogate marker of gastrointestinal dysfunction.

### Secondary endpoints

For both patients, secondary endpoints included change from baseline at 12 and 24 months after ASO in behavioral assessments, including the ORCA and the Aberrant Behavior Checklist. GSV scores were not necessary for the ORCA, as it was initially designed for patients with neurodevelopmental disorders.

The Vineland-3 and BSID-4 with GSVs were also assessed as secondary neurodevelopmental endpoints in Patient 1.

Additional secondary assessments for Patient 1 included the Repetitive Behavior Scale-Revised and Short Sensory Profile Version 2.

### Reporting summary

Further information on research design is available in the Nature Portfolio Reporting Summary linked to this article.

## Online content

Any methods, additional references, Nature Portfolio reporting summaries, source data, extended data, supplementary information, acknowledgements, peer review information; details of author contributions and competing interests; and statements of data and code availability are available at 10.1038/s41591-026-04527-y.

## Supplementary information

Reporting Summary

## Data Availability

Access to the clinical data in this paper is not openly available due to reasons of sensitivity, but may be requested from the corresponding author 1 year from publication by qualified researchers with a response within 90 days. Data will be provided following review and approval of a research proposal and execution of a data use agreement.

## Code availability

The EEG analysis was performed in Python (v.3.14) and is made publicly available at https://github.com/ungTNL/scn2aEEG.

## References

[1] Fisher, R. S. et al. ILAE official report: a practical clinical definition of epilepsy. *Epilepsia* **55**, 475–482 (2014). 24730690 10.1111/epi.12550

[2] Scheffer, I. E. et al. Developmental and epileptic encephalopathies. *Nat. Rev. Dis. Primers* **10**, 61 (2024). 39237642 10.1038/s41572-024-00546-6

[3] Vikin, T. et al. Incidence of childhood and youth epilepsy: a population-based prospective cohort study utilizing current International League Against Epilepsy classifications for seizures, syndromes, and etiologies. *Epilepsia* **66**, 776–789 (2025). 39797741 10.1111/epi.18238 PMC11908673

[4] George, A. L. Jr. Lessons learned from genetic testing for channelopathies. *Lancet Neurol.* **13**, 1068–1070 (2014). 25316008 10.1016/S1474-4422(14)70123-1

[5] Hedrich, U. B. S., Lauxmann, S. & Lerche, H. *SCN2A* channelopathies: mechanisms and models. *Epilepsia* **60**, S68–S76 (2019). 31904120 10.1111/epi.14731

[6] Ganguly, S., Thompson, C. H. & George, A. L. Jr. Enhanced slow inactivation contributes to dysfunction of a recurrent *SCN2A* mutation associated with developmental and epileptic encephalopathy. *J. Physiol.* **599**, 4375–4388 (2021). 34287911 10.1113/JP281834 PMC8446326

[7] Berg, A. T. et al. Expanded clinical phenotype spectrum correlates with variant function in *SCN2A*-related disorders. *Brain* **147**, 2761–2774 (2024). 38651838 10.1093/brain/awae125 PMC11292900

[8] Li, M. et al. Antisense oligonucleotide therapy reduces seizures and extends life span in an *SCN2A* gain-of-function epilepsy model. *J. Clin. Invest.* **131**, e152079 (2021). 34850743 10.1172/JCI152079 PMC8631599

[9] Wagner, M. et al. Antisense oligonucleotide treatment in a preterm infant with early-onset SCN2A developmental and epileptic encephalopathy. *Nat. Med.* **31**, 2174–2178 (2025). 40263630 10.1038/s41591-025-03656-0 PMC12283366

[10] Carroll, J. B. et al. Potent and selective antisense oligonucleotides targeting single-nucleotide polymorphisms in the Huntington disease gene/allele-specific silencing of mutant huntingtin. *Mol. Ther.* **19**, 2178–2185 (2011). 21971427 10.1038/mt.2011.201 PMC3242664

[11] Shomer, I. et al. Personalized allele-specific antisense oligonucleotides for GNAO1-neurodevelopmental disorder. *Mol. Ther. Nucleic Acids.* **36**, 102432 (2024). 39897576 10.1016/j.omtn.2024.102432 PMC11787015

[12] Eltokhi, A. et al. Pathogenic gating pore current conducted by autism-related mutations in the Na_V_1.2 brain sodium channel. *Proc. Natl Acad. Sci. USA.* **121**, e2317769121 (2024). 38564633 10.1073/pnas.2317769121 PMC11009634

[13] Kim-McManus, O. et al. Scaling haplospecific antisense oligonucleotides from N-of-1 to broad use in genetic disease populations by diplotyping. Preprint at *medRxiv*10.64898/2026.01.28.26345012 (2026).

[14] Hipp, J. F. et al. The *UBE3A-*ATS antisense oligonucleotide rugonersen in children with Angelman syndrome: a phase 1 trial. *Nat. Med.* **31**, 2936–2945 (2025). 40646322 10.1038/s41591-025-03784-7

[15] Kim-McManus, O. et al. A framework for N-of-1 trials of individualized gene-targeted therapies for genetic diseases. *Nat. Commun.* **15**, 9802 (2024). 39532857 10.1038/s41467-024-54077-5 PMC11557703

[16] Crooke, S. T., Witztum, J. L., Bennett, C. F. & Baker, B. F. RNA-targeted therapeutics. *Cell Metab.* **27**, 714–739 (2018). 29617640 10.1016/j.cmet.2018.03.004

[17] Wu, H. et al. Determination of the role of the human RNase H1 in the pharmacology of DNA-like antisense drugs. *J. Biol. Chem.* **279**, 1781–1789 (2004). 10.1074/jbc.M311683200 14960586

[18] Pollak, A. J. et al. Inflammatory non-CpG antisense oligonucleotides are signaling through TLR9 in human Burkitt lymphoma B Bjab cells. *Nucleic Acid Ther.* **32**, 473–485 (2022). 36355073 10.1089/nat.2022.0034

[19] O’Rourke J. G. et al. Reversable acute sedation response of phosphorothioate antisense oligonucleotides following local delivery to the central nervous system. Preprint at *bioRxiv*10.1101/2025.02.13.638136 (2025).

[20] Bravo-Hernandez, M. et al. Transient acute neuronal activation response caused by high concentrations of oligonucleotides in the cerebral spinal fluid. *Nucleic Acids Res.* **54**, gkag057 (2026). 41633500 10.1093/nar/gkag057 PMC12867516

