# Supplementary material for: Individualized antisense oligonucleotides for SCN2A-related developmental epileptic encephalopathy
Source: Nat Med. 2026 Jul 21;32(8):2838–45. doi: 10.1038/s41591-026-04527-y (PMC13472884; doi:10.1038/s41591-026-04527-y)
Supplement: Supplementary file 1 [file 41591_2026_4527_MOESM1_ESM.pdf]

nature portfolio

Corresponding author(s): Olivia Kim-McManus

Last updated by author(s): May 26, 2026

Reporting Summary

Nature Portfolio wishes to improve the reproducibility of the work that we publish. This form provides structure for consistency and transparency in reporting. For further information on Nature Portfolio policies, see our Editorial Policies and the Editorial Policy Checklist.

Statistics

For all statistical analyses, confirm that the following items are present in the figure legend, table legend, main text, or Methods section.

n/a	Confirmed	
☐	☒	The exact sample size (*n*) for each experimental group/condition, given as a discrete number and unit of measurement
☐	☒	A statement on whether measurements were taken from distinct samples or whether the same sample was measured repeatedly
☐	☒	The statistical test(s) used AND whether they are one- or two-sided<br>*Only common tests should be described solely by name; describe more complex techniques in the Methods section.*
☐	☒	A description of all covariates tested
☒	☐	A description of any assumptions or corrections, such as tests of normality and adjustment for multiple comparisons
☐	☒	A full description of the statistical parameters including central tendency (e.g. means) or other basic estimates (e.g. regression coefficient) AND variation (e.g. standard deviation) or associated estimates of uncertainty (e.g. confidence intervals)
☐	☒	For null hypothesis testing, the test statistic (e.g. *F*, *t*, *r*) with confidence intervals, effect sizes, degrees of freedom and *P* value noted<br>*Give P values as exact values whenever suitable.*
☒	☐	For Bayesian analysis, information on the choice of priors and Markov chain Monte Carlo settings
☒	☐	For hierarchical and complex designs, identification of the appropriate level for tests and full reporting of outcomes
☒	☐	Estimates of effect sizes (e.g. Cohen's *d*, Pearson's *r*), indicating how they were calculated

Our web collection on statistics for biologists contains articles on many of the points above.

Software and code

Policy information about availability of computer code

Data collection	*Provide a description of all commercial, open source and custom code used to collect the data in this study, specifying the version used OR state that no software was used.*
Data analysis	EEG analysis as described in Methods and Supplementary materials was performed in Python and is made publicly available at: https://github.com/ungTNL/scn2aEEG

For manuscripts utilizing custom algorithms or software that are central to the research but not yet described in published literature, software must be made available to editors and reviewers. We strongly encourage code deposition in a community repository (e.g. GitHub). See the Nature Portfolio guidelines for submitting code & software for further information.

Data

Policy information about availability of data

All manuscripts must include a data availability statement. This statement should provide the following information, where applicable:

- Accession codes, unique identifiers, or web links for publicly available datasets
- A description of any restrictions on data availability
- For clinical datasets or third party data, please ensure that the statement adheres to our policy

All data supporting findings of this study are provided within the manuscript. Complete protocols are provided in full. Access to the clinical data in this paper may

be requested from the corresponding author after one year from publication by qualified researchers with response provided within 90 days. Data will be provided following review and approval of a research proposal and execution of a Data Sharing agreement

Research involving human participants, their data, or biological material

Policy information about studies with human participants or human data. See also policy information about sex, gender (identity/presentation), and sexual orientation and race, ethnicity and racism.

Reporting on sex and gender	Age and sex are reported. Gender based analyses were unable to be performed. Study consisted of two nonverbal pediatric patients with cognitive impairment and severe neurodevelopmental delay.
Reporting on race, ethnicity, or other socially relevant groupings	This is a rare genetic disease which presents in neonates and children who may not survive into adulthood. There are practical limitations to broad demographic reporting from two parallel n=1 single participant studies of rare genetic disease. Due to extreme disease rarity and medical complexity of SCN2A variants, it is not possible to broadly report on race, ethnicity or other socially relevant groupings in this study as typically outlined for standard clinical trials with larger cohorts.
Population characteristics	*Describe the covariate-relevant population characteristics of the human research participants (e.g. age, genotypic information, past and current diagnosis and treatment categories). If you filled out the behavioural & social sciences study design questions and have nothing to add here, write "See above."*
Recruitment	Study participants included pediatric patients with known SCN2A developmental epileptic encephalopathy due to rare causal variants, intractable epilepsy, autism spectrum disorder, and severe neurodevelopmental delay.
Ethics oversight	The studies were approved by each respective institutional review board and ethical experimental treatment oversight committees.

Note that full information on the approval of the study protocol must also be provided in the manuscript.

Field-specific reporting

Please select the one below that is the best fit for your research. If you are not sure, read the appropriate sections before making your selection.

☒ Life sciences ☐ Behavioural & social sciences ☐ Ecological, evolutionary & environmental sciences

For a reference copy of the document with all sections, see nature.com/documents/nr-reporting-summary-flat.pdf

Life sciences study design

All studies must disclose on these points even when the disclosure is negative.

Sample size	Research investigational new drug (INDs) applications were authorized by the FDA for two investigator-initiated, open-label, single center, single patient (n=1) clinical trials for distinct pathogenic SRD variants with predefined safety and efficacy measures tailored to individual phenotype. The allele-selective ASOs were delivered intrathecally by lumbar injection in these two first-in-human clinical trials.
Data exclusions	There were no data exclusions.
Replication	Dosing schema as authorized by the FDA included replication of doses within individual participants.
Randomization	Randomization is not applicable by study design, which included open label single participants at each academic institution.
Blinding	The study design was open label as authorized by the FDA.

Reporting for specific materials, systems and methods

We require information from authors about some types of materials, experimental systems and methods used in many studies. Here, indicate whether each material, system or method listed is relevant to your study. If you are not sure if a list item applies to your research, read the appropriate section before selecting a response.

Materials & experimental systems

n/a	Involved in the study
☒	☐ Antibodies
☒	☐ Eukaryotic cell lines
☒	☐ Palaeontology and archaeology
☒	☐ Animals and other organisms
☐	☒ Clinical data
☒	☐ Dual use research of concern
☒	☐ Plants

Methods

n/a	Involved in the study
☒	☐ ChIP-seq
☒	☐ Flow cytometry
☒	☐ MRI-based neuroimaging

Clinical data

Policy information about clinical studies

All manuscripts should comply with the ICMJE guidelines for publication of clinical research and a completed CONSORT checklist must be included with all submissions.

Clinical trial registration: NCT06314490

Study protocol: Study protocols in full are included as part of manuscript submission.

Data collection: Data collection was completed at each academic institution from trial participants as specified by protocols during the trial.

Outcomes: Outcomes were predefined and selected tailored to phenotype

Plants

Seed stocks: N/A

Novel plant genotypes: N/A

Authentication: N/A
